# Deregulation of RNAi silencing pathway by Human T-cell leukemia virus type 1 bZIP factor (HBZ)

**DOI:** 10.1186/1742-4690-11-S1-O55

**Published:** 2014-01-07

**Authors:** Hélène Gazon, Jean-Michel Mesnard, Raymond Césaire, Jean-Marie Peloponese

**Affiliations:** 1 CPBS, CNRS UMR 5236, Montpellier, France; 2 CPBS, Université Montpellier 1, Montpellier, France; 3 Laboratoire de Virologie-Immunologie, Centre Hospitalier et Universitaire de Fort de France, Fort de France, Martinique, France

## 

Human T-cell leukemia virus type-1 (HTLV-1) is the causative agent of a non-Hodgkin leukemia known as adult T cell leukemia (ATL). MicroRNAs (miRNAs) are a class of non-coding RNAs that regulate the expression of target genes at posttranscriptional level. Biogenesis of miRNAs is tightly regulated and starts with the production of pri-miRNAs by RNA polymerase II. The pri-miRNAs are then cleaved by Drosha in conjunction with DGCR8 in the nucleus, generating pre-miRNAs, which are then transferred to the cytoplasm where Dicer1 further processes the hairpin into the mature miRNAs. With exception of few types of neoplasm, a general downregulation of miRNAs expression has been observed in cancers cells, mainly due to chromosomal abnormalities, epigenetic changes or aberrant expression of the miRNA biogenesis factors. Involvement of miRNAs alteration in the HTLV-1 life cycle has recently come to light. However, the mechanisms that regulate the expression of miRNAs biogenesis factors in ATL cells are still largely unknown. In this study, we assessed Dicer1 expression levels in ATL patients and investigated its transcriptional regulation in HTLV infected cell lines. We reported a significant correlation between reduced miRNA biogenesis and expression of HTLV-1 basic leucine zipper factor (HBZ). We showed that Dicer1 expression was downregulated in ATL patients vs. asymptomatic patients by qRT-PCR and western blot analysis. We also demonstrated that Dicer1 was regulated by AP-1 factors, through luciferase and Chip assays. Our data suggest that Dicer1 expression may be used as a promising prognostic marker and therapeutic target for ATL.

